# Contrasts in niche breadth and its drivers between soil microbes and plants in alpine grasslands

**DOI:** 10.1093/ismeco/ycag193

**Published:** 2026-07-09

**Authors:** Lei Chen, Ke Dong, Mengchen Zhao, Zhi Yu, Nan Li, Dorsaf Kerfahi, Yu Shi, Xin Jing, Litong Chen, Haiyan Chu, Jinsheng He, Sang-Seob Lee, Teng Yang, Jonathan M Adams

**Affiliations:** Department of Integrative Biotechnology, Sungkyunkwan University, Suwon 16419, Republic of Korea; Department of Biological Sciences, Kyonggi University, Suwon 16227, Republic of Korea; Department of Integrative Biotechnology, Sungkyunkwan University, Suwon 16419, Republic of Korea; Department of Environmental Health Sciences, Graduate School of Public Health, Seoul National University, Seoul 08826, Republic of Korea; Key Laboratory of Climate, Resources and Environment in Continental Shelf Sea and Deep Sea of Department of Education of Guangdong Province, Department of Oceanography, Key Laboratory for Coastal Ocean Variation and Disaster Prediction, College of Ocean and Meteorology, Guangdong Ocean University, Zhanjiang 524088, China; Department of Biological Sciences, Department of Natural Sciences, Keimyung University, Daegu 42601, Republic of Korea; State Key Laboratory of Crop Stress Adaptation and Improvement, School of Life Sciences, Henan University, Kaifeng 475004, China; State Key Laboratory of Herbage Improvement and Grassland Agro-Ecosystems and College of Pastoral Agriculture Science and Technology, Lanzhou University, Lanzhou 730000, China; Qinghai Haibei National Field Research Station of Alpine Grassland Ecosystem, Northwest Institute of Plateau Biology, Chinese Academy of Sciences, Xining 810008, China; State Key Laboratory of Soil and Sustainable Agriculture, Institute of Soil Science, Chinese Academy of Sciences, Nanjing 211135, China; University of Chinese Academy of Sciences, Beijing 101408, China; Institute of Ecology, College of Urban and Environmental Sciences, and Key Laboratory for Earth Surface Processes of the Ministry of Education, Peking University, Beijing 100871, China; Department of Integrative Biotechnology, Sungkyunkwan University, Suwon 16419, Republic of Korea; State Key Laboratory of Soil and Sustainable Agriculture, Institute of Soil Science, Chinese Academy of Sciences, Nanjing 211135, China; University of Chinese Academy of Sciences, Beijing 101408, China; University of Chinese Academy of Sciences, Nanjing 211135, China; School of Geography and Ocean Science, Nanjing University, Nanjing 210023, China

**Keywords:** niche breadth, biotic and abiotic factors, elevational gradient, Qinghai–Tibet plateau

## Abstract

Niche breadth reflects the extent to which taxa or communities can tolerate environmental variations, indicating their potential to respond to environmental changes. However, it remains unclear whether micro- and macro-organisms share similar patterns of niche breadth along the same environmental gradients, and which environmental filters regulates niche breadth across different trophic levels. In this study, 180 soil samples were collected from the northeastern and central Qinghai–Tibet Plateau. By integrating microbiome sequencing with plant community surveys, we quantified niche breadth for bacteria, fungi, and plants within a multidimensional environmental space defined by principal component analysis of environmental variables. We then compared niche breadth among the three groups and identified their primary environmental drivers. The results showed that fungal communities exhibited the broadest niche breadth overall, whereas plant communities displayed the greatest variability in mean niche breadth amongst sites. Elevation was a shared determinant of niche breadth in all groups, although with contrasting response patterns. Bacterial and fungal communities exhibited broader niche breadths at higher elevations, whereas plant communities reached their maximum niche breadths at mid-elevations, suggesting contrasting adaptive strategies. A combination of abiotic (geography and environment) and biotic (community diversity and composition) factors better explained the variation in niche breadth. In particular, compared with the niche breadth of fungal communities, that of bacterial and plant communities was more strongly regulated by biotic factors such as community composition. We further confirmed that abiotic factors influenced the niche breadth of the three biological groups, both directly and indirectly, by shaping their biotic attributes. From the perspective of niche breadth, this study revealed distinct environmental adaptation patterns and the underlying mechanisms of different biological groups in alpine grassland ecosystems. Better understanding of niche breadth patterns amongst different kingdoms of life may provide improved prediction of turnover and resilience of soil ecosystems under environmental change.

## Introduction

Niche breadth refers to the range of environmental conditions and resources that a species can tolerate or utilize, and is widely regarded as an important indicator of ecological adaptability and competitive capacity within ecosystems, as well as of resource-use efficiency and ecosystem stability [1–4]. Species with broader niche breadths tend to persist across diverse environmental settings, while those with narrower niches are typically more specialized and potentially more sensitive to environmental change [5, 6]. At the community level, niche breadth reflects the aggregate ecological strategies of coexisting taxa and can inform assessments of resource-use complementarity, ecosystem stability, and resilience to disturbance [7, 8]. In terrestrial ecosystems, plants and soil microorganisms, including bacteria and fungi, interact closely to regulate key ecological processes such as organic matter decomposition, plant–soil feedback, and biogeochemical cycling [9, 10]. Plants modify the availability and spatial heterogeneity of soil resources through root exudates, litter inputs, and nutrient uptake, whereas soil microorganisms influence plant growth and environmental adaptation through decomposition, nutrient cycling, and symbiotic or antagonistic interactions [10, 11]. Because these groups coexist under the same broad gradients in climate and soil conditions, their niche breadth can be evaluated within a shared environmental framework despite differences in life history, dispersal, and resource use. However, most empirical studies have focused on niche breadth variation within single taxonomic groups or along isolated environmental gradients [6, 12–14], leaving cross-kingdom variation in niche strategies under shared environmental conditions poorly understood.

As an integrative measure of resource-use capacity, niche breadth reflects how broadly species or communities utilize environmental resources and is therefore closely linked to the influence of environmental filtering on community structure and ecosystem functioning [15, 16], and niche breadth is influenced by a variety of abiotic factors. Soil physicochemical properties—such as pH, organic matter content, C:N ratio, salinity, and heavy metal concentrations—are known to shape microbial niche breadth by constraining resource availability and microbial metabolic flexibility [4, 17–20]. In nutrient-poor or physiochemically stressful soils (e.g. low pH, high salinity), microbial communities often exhibit narrower niche breadths due to reduced metabolic potential and substrate access [7]. Similar effects have been observed in plant communities, where niche breadth is closely linked to soil nutrient availability, particularly nitrogen and phosphorus [21], which influence spatial resource partitioning and species coexistence. In addition, climatic factors—such as temperature and precipitation—act as dominant environmental filters, governing species survival, distribution, and niche expression [22–24]. For instance, drought stress has been shown to constrain microbial niche breadth and promote ecological specialization [25], while alpine and subalpine plant communities display strong climate-dependent distributional patterns [26]. At larger spatial scales, geographic factors, including topographic barriers, can restrict dispersal and gene flow, thereby narrowing realized niches [27, 28]. Furthermore, macroecological studies have suggested a latitudinal gradient in niche breadth, with lower-latitude communities often characterized by narrower niches due to stable but competitive environments [5, 29].

While abiotic filters are key drivers of niche breadth, biotic factors—such as species diversity, community composition, and interspecific interactions—also play fundamental roles [30–34]. High community diversity can promote resource partitioning and ecological specialization, thereby reducing niche overlap and overall niche breadth [35–38]. Interspecific relationships, including mutualism, competition, and antagonism, can reshape resource-use strategies, alter spatial structure, and influence the breadth of niches occupied [39, 40]. For example, bacterial succession during colonization can result in dominant taxa expanding their niche breadth under increasing competitive pressure [41]. Similarly, in tropical plant communities, rising species richness and temperature have been linked to intensified competition and reduced niche breadth [42]. Importantly, biotic effects are often context-dependent and interact with abiotic constraints [43]. Geographical isolation may intensify local environmental filtering, promote niche differentiation, or enhance niche overlap among microbial taxa [44]. Moreover, climate-driven increases in temperature or moisture can enhance diversity and interspecific competition, potentially narrowing niche breadth [45, 46], while long-term climatic stability may foster ecological specialization through persistent community assembly processes [47]. However, in terrestrial ecosystems, the formation of niche breadth is not solely determined by within-community processes, and interactions across different biological kingdoms may also play a key role [48]. In particular, within plant–soil microbial systems, plant communities can modify the availability and spatial heterogeneity of soil resources through root exudation, litter inputs, and nutrient uptake, thereby influencing the resource-use range of bacteria and fungi [49]. Conversely, soil bacteria and fungi can regulate plant resource acquisition and environmental adaptation by mediating organic matter decomposition, nutrient cycling, pathogen suppression, and symbiotic interactions [50, 51]. Taken together, these insights underscore the need for an integrative framework that accounts for both abiotic and biotic drivers of niche breadth. Despite growing interest in understanding niche-based processes, comparative investigations across distinct biological kingdoms—particularly under shared environmental contexts—remain limited. A unified perspective that bridges microbial and plant ecology is essential for uncovering general principles of niche differentiation and for predicting how complex communities respond to global environmental change.

The Qinghai–Tibet Plateau, the world’s largest alpine ecosystem, provides a natural laboratory for investigating ecological adaptation and its drivers due to its broad environmental gradients [52]. In this study, we analysed 180 soil samples collected from the northeastern and central Tibetan Plateau regions and assessed niche breadths of bacteria, fungi, and plants using plant community surveys and amplicon sequencing of bacterial 16S rRNA genes and fungal internal transcribed spacer (ITS) regions. The dataset we analysed is particularly suitable for comparison of niches because it includes a large number of standard samples where vegetation species composition, bacterial soil community, and fungal soil community were sampled together along very broad environmental gradients. Here, we propose the following hypotheses: (i) Due to fundamental differences in life history traits, dispersal capacity, and physiological plasticity, bacteria, fungi, and plants differ significantly in their niche breadths. (ii) Despite being influenced by the same set of abiotic environmental gradients (e.g. pH, moisture, elevation), their responses along environmental gradients differ. (iii) Niche breadth is determined by the interplay between abiotic constraints and biotic interactions, with the relative influence of each varying among microbial and plant communities.

## Materials and methods

### Sample collection

**Figure 1:**
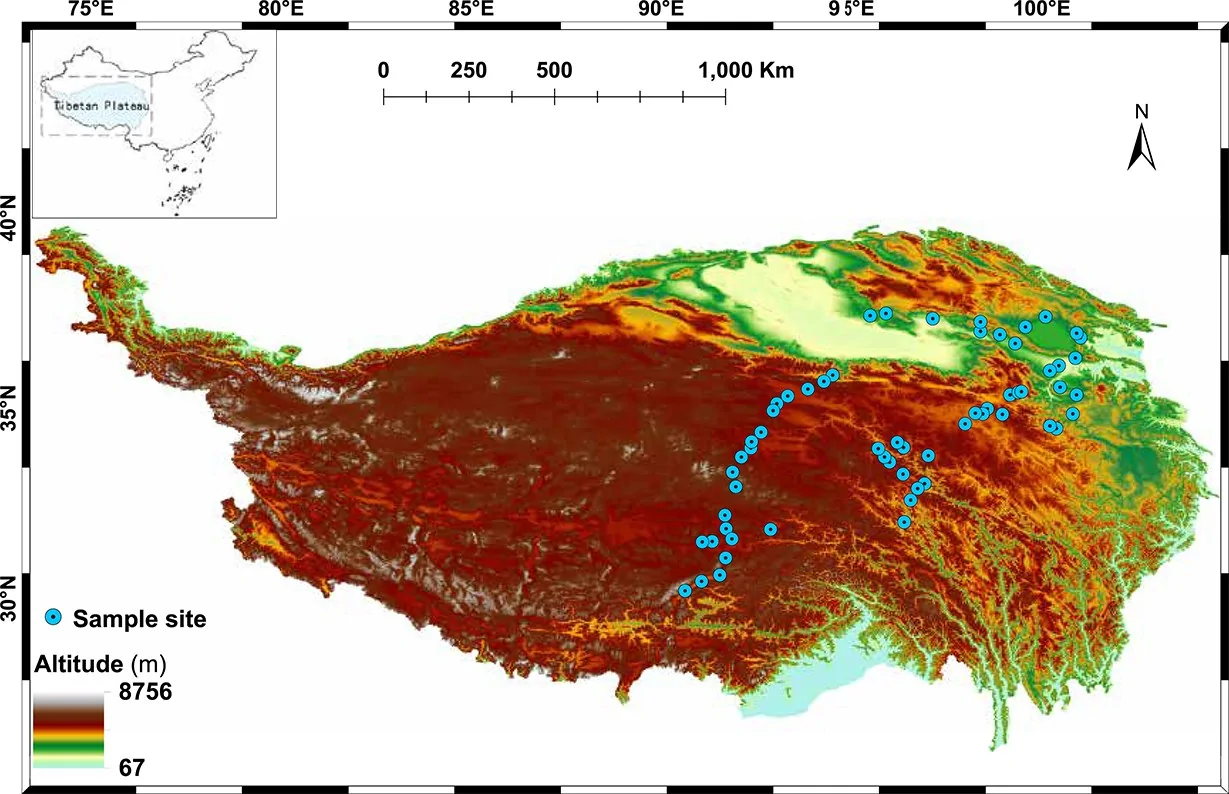
Map of 60 sampling sites across the Qinghai–Tibet Plateau. Each site consisted of three independent 1 × 1 m plots used as the minimum replicates.

A total of 60 alpine grassland sites, comprising 180 individual quadrat samples, were surveyed across the northeastern and central regions of the Qinghai–Tibet Plateau, spanning ~815 km north–south and 960 km east–west (Fig. 1). The study area was characterized by high environmental heterogeneity, encompassing diverse vegetation types (alpine meadows, alpine steppe, and desert steppe), broad climatic gradients (mean annual temperature ranging from −5.2°C to 4.7°C and mean annual precipitation from 66 to 560 mm), an elevational range of 2918–5228 m, and substantial variation in soil physicochemical properties. Sampling was conducted during the peak growing season (July–August 2011) to capture peak plant and microbial activity. At each 100 m × 100 m site, three 1 m × 1 m plots were randomly established along the diagonal, with a minimum spacing of 40 m between plots to reduce spatial autocorrelation within each site [53], community compositional dissimilarity was greater between sites than within sites (Table S1). Plant cover and species composition were recorded (Table S2). Seven soil cores (5 cm diameter) were randomly collected from each plot. After removing the surface litter, the 0–5 cm topsoil was homogenized in the field to obtain a representative composite sample. Soil samples were sieved through a 2 mm mesh to remove visible roots and litter and then split into two portions. One part was stored at 4°C for soil property analysis, and the other was stored at −80°C for DNA extraction and microbial community analysis.

### Vegetation survey and measurements of climate and soil properties

All vascular plant species in each of the 180 plots were identified and counted in the field (Table S2). Across all recorded plant species, forbs, sedges, grasses, and legumes accounted for 44.1%, 29.1%, 21.6%, and 5.0%, respectively. Soil physicochemical properties were measured at the site level and averaged across plots. The soil pH was measured in a 1:5 (w/v) soil-to-water slurry using a pH meter (Thermo Orion-868, Thermo Orion Co., Waltham, MA, USA). Soil moisture (SM) was determined gravimetrically. Prior to analysis, air-dried soil samples were ground and passed through a 100-mesh sieve after removing visible plant residues and gravel. Total carbon (TC) and total nitrogen (TN) were measured using an elemental analyser (2400 II CHN, PerkinElmer, Boston, MA, USA). For dissolved TN (DTN), ~5 g of fresh soil was extracted with 50 ml of 2 mol L^−1^ KCl solution by shaking for 1 h, followed by settling for ~30 min and filtration. For dissolved organic carbon (DOC), ~5 g of fresh soil was extracted with 50 ml of deionized water by shaking for 1 h, followed by settling for ~30 min. The supernatant was centrifuged for 5 min at 4000 rpm and then filtered. The filtrates (20–30 ml) were transferred into 50 ml tubes and stored frozen prior to analysis. DOC and DTN (including NH₄^+^-N, NO₃^−^-N, and dissolved organic N) concentrations were quantified using a total organic carbon/total nitrogen (TOC/TN) analyser (Shimadzu, Japan). Soil total phosphorus (STP) was determined by the molybdenum blue method with an ultraviolet-visible spectrophotometer (UV-2550; Shimadzu, Kyoto, Japan). Soil inorganic carbon (SIC) was calculated from the carbonate content using calcimetry (Eijkelkamp, Giesbeek, the Netherlands), and soil organic carbon (SOC) was obtained by subtracting SIC from TC. Mean annual temperature (MAT) and mean annual precipitation (MAP) were estimated by interpolating data from 716 meteorological stations across China between 1951 and 2010 (source: http://cdc.cma.gov.cn), following the method described by Jing et al. [54]. Vegetation productivity was represented by the normalized difference vegetation index (NDVI) derived from MODIS (Terra satellite; 250 m resolution, 16-day composites during July–August) obtained from NASA (https://ladsweb.nascom.nasa.gov/data/search.html).

The study area exhibited broad climatic and edaphic heterogeneity across sampling sites (Table S3). MAT ranged from −5.18°C to 4.67°C, and MAP ranged from 66.45 to 560.17 mm. Soil pH ranged from 6.03 to 9.15, while SM varied from 0.003 to 1.537 g g^−1^. Nutrient-related variables also showed substantial variation, with TC ranging from 0.72 to 21.95 g kg^−1^, TN from 0.02 to 1.66 g kg^−1^, and C:N from 9.09 to 92.50. Detailed descriptions and ranges of all environmental variables are provided in Table S3.

### Sequencing and bioinformatics

Total DNA was extracted from 0.5 g of air-dried soil using the FastDNA® Spin Kit (Bio 101, Carlsbad, CA, USA) according to the manufacturer’s instructions. For bacterial communities, the V4 region of the 16S rRNA gene was amplified using the primers 515F/806R [55], following the protocol described by Jing *et al*. [54]. For fungal communities, the ITS region was amplified using the primer pair ITS3/ITS4 as described by Yang *et al*. [56]. Sequencing was performed using the Illumina MiSeq PE250 platform (Illumina Inc., San Diego, CA, USA).

The paired-end reads were merged using FLASH [57]. Sequence quality filtering, trimming, and chimaera removal were conducted using QIIME v1.9.0 [58]. Bacterial taxonomic annotation was performed using the SILVA SSU release 138 database (https://www.arb-silva.de/) [59], whereas fungal taxa were assigned using BLASTn against the UNITE v9.0 database (http://unite.ut.ee) [60]. High-quality sequences were clustered into operational taxonomic units (OTUs) at 97% similarity using the Usearch algorithm [61]. After quality filtering, the average sequencing depth was 5148 reads per sample for bacterial 16S rRNA genes and 64 313 reads per sample for fungal ITS sequences. To ensure comparability among samples and minimize biases caused by uneven sequencing depth, the feature tables were rarefied to the minimum sequencing depth across samples, resulting in 3502 reads per sample for bacteria (Table S4) and 11 460 reads per sample for fungi (Table S5).

### Community diversity, composition, and niche breadth calculation

The alpha diversity (Shannon index) of bacterial, fungal, and plant communities was calculated using the diversity function in the R package vegan (version 4.1.3). Bray–Curtis dissimilarities were computed using the phyloseq and vegan packages, and nonmetric multidimensional scaling (NMDS) was performed based on these distances. The first axis score (NMDS1) was extracted as an indicator of community compositional differences (stress values: bacteria = 0.126, fungi = 0.165, and plants = 0.0002) and was calculated using the metaMDS function in vegan (Table S6).

To quantify environmental niche breadth, we constructed a multidimensional environmental space based on 15 measured environmental variables across 180 sampling plots. Principal component analysis (PCA) was performed using the rda function in the vegan package, and the first 10 principal components, which explained 96.99% of the total environmental variation (Fig. S1 and Table  S7) were retained for subsequent analyses. OTUs occurring in fewer than three sampling sites were excluded prior to niche analyses to avoid unstable estimates caused by extremely low occurrence.

Niche breadth for each OTU was calculated using the ecospat.nichePOSNB function in the ecospat package [62]. Overall niche breadth was then calculated using the ecospat.nicheNBmean function [13, 62], as the weighted mean across PCA axes with weights corresponding to the variance explained by each axis (Tables S8–S10):


$$ {B}_i=\sum \left({w}_k\times N{B}_{ik}\right) $$

where ${B}_i$is the overall niche breadth of OTU *i*, $N{B}_{ik}$is its niche breadth along PCA axis *k*, and ${w}_k$represents the proportion of variance explained by axis *k*.

Community-level niche breadth was calculated for each sample as the mean niche breadth of all occurring OTUs (Table S11):


$$ {B}_{comm}=\frac{\sum \left({I}_i\times{B}_i\right)}{\sum{I}_i} $$

where ${I}_i$indicates whether OTU *i*is present in a given sample (1 = present, 0 = absent), and ${B}_i$is the overall niche breadth of that OTU. This presence–absence-based approach was used to characterize the average niche strategy within each community while reducing the influence of highly abundant taxa. Differences among groups were assessed using one-way ANOVA followed by Bonferroni *post hoc* tests, and figures were generated using the ggplot2 package in R.

### Statistical analyses

A Spearman rank correlation analysis was performed to examine the relationships between environmental variables and niche breadth, using the cor.test() function from the *stats* package in R. To further evaluate the relative importance of predictor variables, a random forest regression model was constructed using the scikit-learn library in Python (version 1.1.3), and variable importance was quantified as the percentage increase in mean squared error (%IncMSE). Statistical significance was assessed using permutation tests by randomly permuting the response variable and repeatedly refitting the random forest model to generate a null distribution, against which the observed %IncMSE values were compared to obtain *P*-values. To disentangle the relative contributions of different types of factors, we categorized all explanatory variables into three groups: (i) geography: longitude and latitude; (ii) environment: elevation, mean annual temperature (MAT), mean annual precipitation (MAP), soil moisture (SM), bulk density (Bulkd), pH, STP, TC, TN, C:N ratio, DOC, SIC, SOC, DTN, and NDVI; (iii) biotic: Shannon index (diversity) and NMDS1 (composition) for bacteria, fungi, and plants. Variation partitioning analysis (VPA) was conducted using the varpart function in the vegan package in R to quantify the independent and joint explanatory powers of the three variable sets.

We constructed a structural equation model (SEM) to explore the direct and indirect effects of environmental, geographic, and biotic factors on niche breadth. The SEM was implemented in AMOS software (version 23.0, IBM, USA). Noncollinear abiotic variables (excluding TC, TN, and SOC) were Z-standardized. Environmental dissimilarity among sites was calculated based on Euclidean distances, whereas geographic dissimilarity was computed using Haversine distances (via the geosphere package). Community compositional dissimilarity (Bray–Curtis) was calculated separately for bacteria, fungi, and plants. The resulting pairwise distance matrices were then integrated by matching sample pairs to generate a combined cross-kingdom dissimilarity dataset. Shannon diversity values for bacteria, fungi, and plants were combined and standardized, and pairwise diversity dissimilarity among samples was then calculated using Manhattan distances. In SEM modelling, we constructed a three-layer path structure based on theoretical assumptions and previous analytical results. The first layer included environmental and geographic dissimilarities; the second layer consisted of differences in community composition and diversity; and the third layer represented differences in niche breadth. Separate SEMs were constructed for bacteria, fungi, and plants to identify taxon-specific pathways through which environmental changes influenced the niche breadth via community composition and diversity. Model fit was assessed based on χ^2^/df, root mean square error of approximation (RMSEA), comparative fit index (CFI), and goodness-of-fit index (GFI). A model was considered well-fitting if CFI and GFI > 0.90, RMSEA <0.08, χ^2^/df < 3, and *P* > .05 [63]. Nonsignificant paths were iteratively removed to optimize the model fit, and the direct, indirect, and total path effects for each variable were reported. SEM analyses were conducted using an AMOS 28.0 (Smallwaters Corporation, Chicago, IL, USA).

## Results

### Cross-kingdom differences in niche breadth

**Figure 2:**
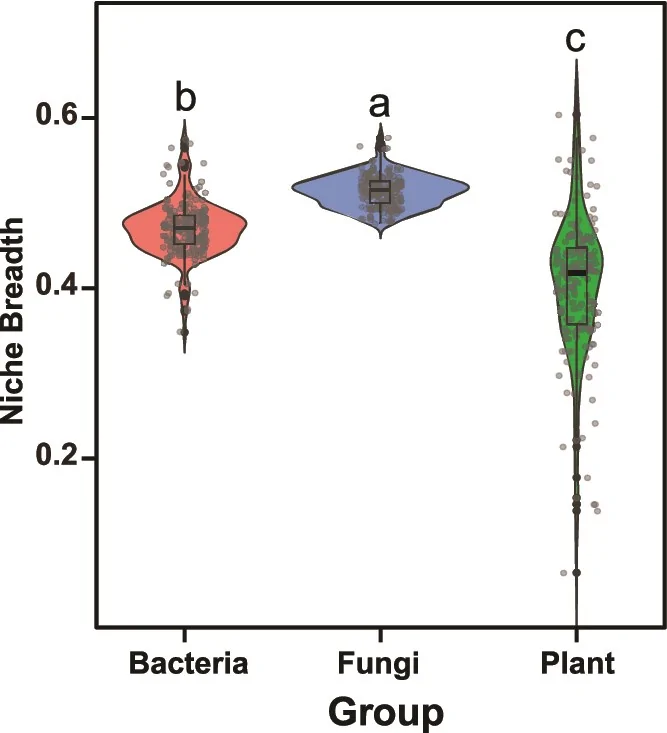
Community niche breadth of bacteria, fungi, and plants. Different letters indicate significant differences between groups (*P* < .05).

We compared niche breadth among soil bacteria, fungi, and plants across 180 plots at both the taxon and community levels. Significant differences among the three groups were observed at both scales (ANOVA, *P* < .001; Fig. S2; Fig. 2). At the community level, fungal communities exhibited the highest mean niche breadth (mean ≈ 0.51), bacterial communities were intermediate (mean ≈ 0.47), and plant communities had the lowest niche breadth (mean ≈ 0.41). The distribution of community-level niche breadth among sites also differed among groups: fungal communities showed relatively low dispersion, whereas plant communities exhibited greater dispersion across plots (Fig. 2).

To further examine taxonomic variation in niche breadth, we compared niche breadth among major bacterial phyla, fungal phyla, and dominant plant genera (Fig. S4). Significant variation in niche breadth was observed among taxonomic groups across all three kingdoms. In bacterial communities, Bacillota and Pseudomonadota generally exhibited relatively broader niche breadths than several other phyla (Fig. S4a). Fungal niche breadth also differed among phyla, with Rozellomycota showing comparatively broader niche breadths than other fungal groups (Fig. S4b). Similarly, dominant plant genera displayed distinct variation in niche breadth, with Kobresia exhibiting relatively broader niche breadths than several other genera (Fig. S4c).

### Abiotic factors affecting community niche breadth

**Figure 3:**
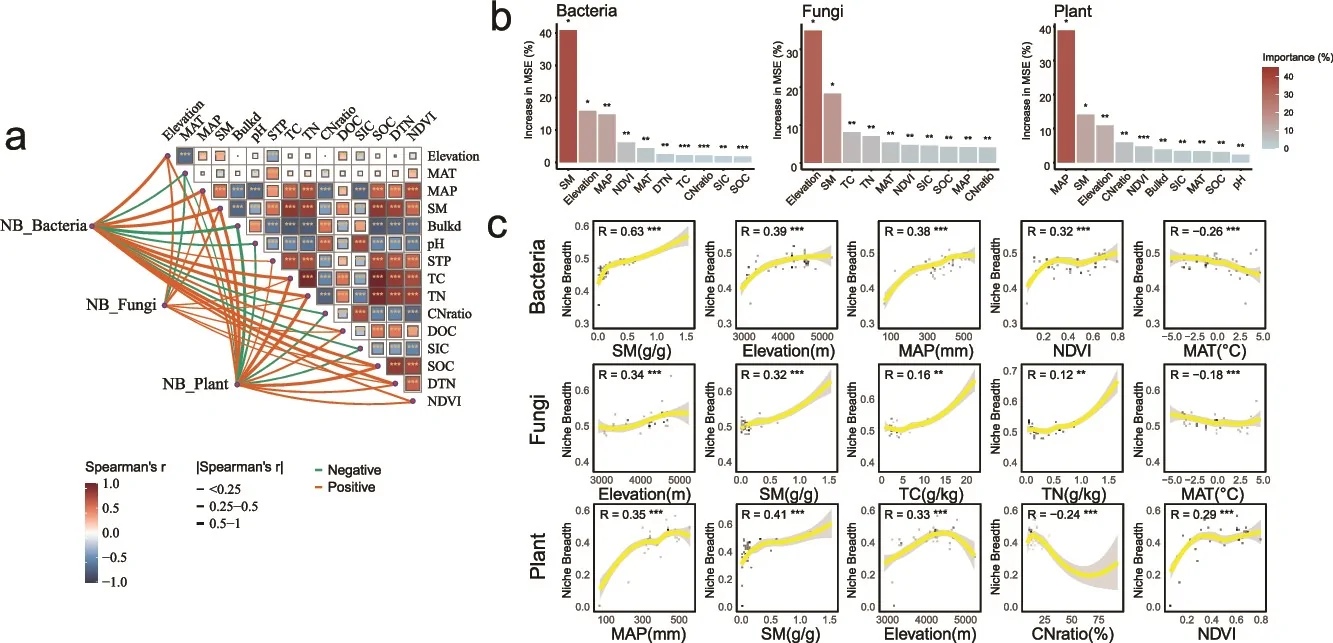
Effects of environmental factors on community niche breadth. (a) Correlation network and matrix of environmental variables significantly affecting the community niche breadth of bacteria, fungi, and plants. (b) Variable importance ranking from random forest analysis for environmental factors influencing ‘community’ niche breadth of bacteria, fungi, and plants. (c) Scatter plots and fitted response curves of the top five important variables vs. community niche breadth for the three groups. Different significance levels are indicated as follows: ^*^P < .05; ^**^P < .01; ^***^P < .001.

The community niche breadths of soil bacteria and plants were significantly correlated with multiple environmental factors (Spearman, *P* < .05; Fig. 3a). Specifically, the bacterial community niche breadth was strongly positively correlated with SM (*r* = 0.63), elevation (*r* = 0.39), and MAP (*r* = 0.38) but negatively correlated with MAT (*r* = −0.26). Plant community niche breadth was mainly positively influenced by MAP (*r* = 0.35), SM (*r* = 0.41), and elevation (*r* = 0.33) but negatively correlated with soil C/N (*r* = −0.24). Fungal community niche breadth was positively correlated with elevation (*r* = 0.34), SM (*r* = 0.32), and TC (*r* = 0.16) but weakly negatively correlated with MAT (*r* = −0.18).

Random forest analysis revealed distinct response patterns among the three groups (Fig. 3b). Bacterial community niche breadth was most strongly influenced by SM, followed by elevation and MAP. Fungal community niche breadth prediction depends primarily on elevation and SM and TC levels. The plant community niche breadth was mainly regulated by MAP, SM, elevation, and C/N. The response curves of the top five important variables in the random forest model showed significant niche breadth responses along environmental gradients for all three groups (Fig. 3c). Notably, along the elevation gradient, bacterial and fungal community niche breadth increased monotonically and peaked at high elevations, whereas plant community niche breadth peaked at mid-elevations, forming a unimodal response pattern. Moreover, the niche breadth of all three communities increased significantly with increasing SM content. Although community-level niche breadth differed significantly in its response to environmental factors among the three groups (interaction, *P* < .01), their dominant drivers differed; microbial communities, especially bacteria, were more dependent on soil factors, whereas plant communities were more driven by climatic factors.

### Combined effects of abiotic and biotic factors on niche breadth

**Figure 4:**
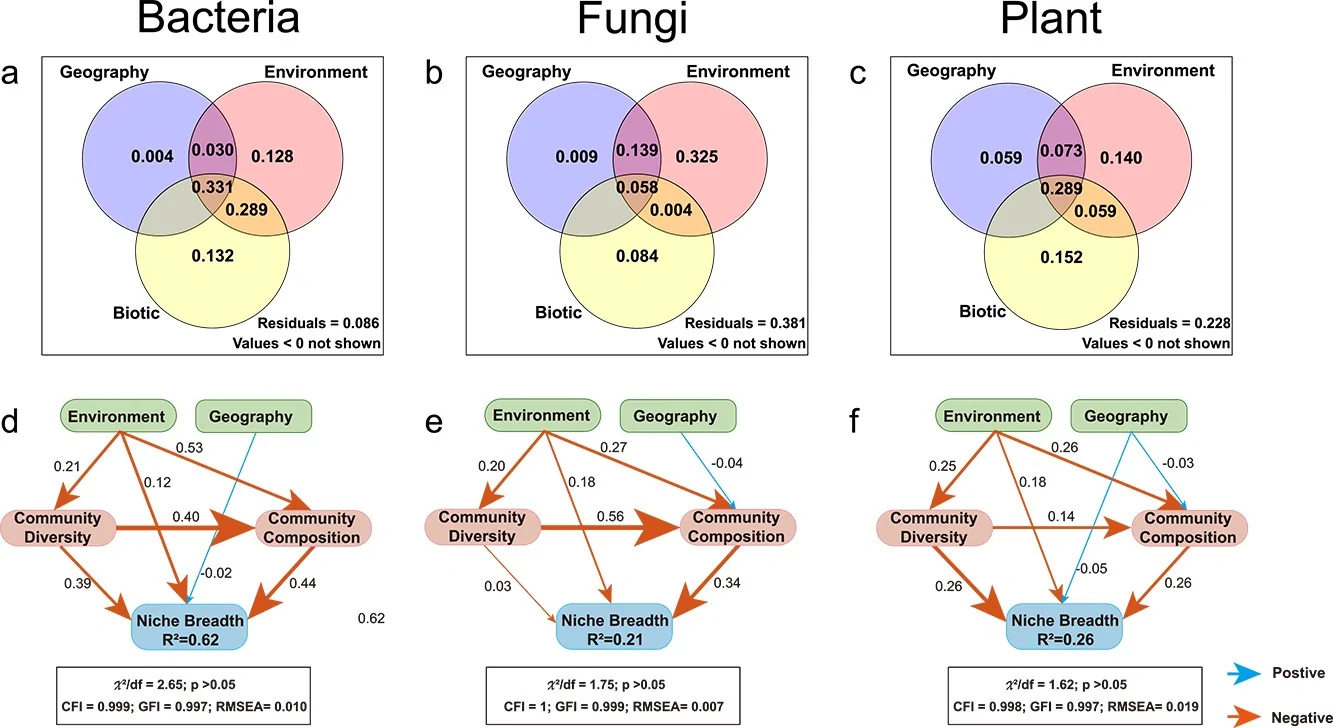
Independent and joint explanatory power of environmental, geographic, and biotic factors on community niche breadth, and SEM of their pathways. Venn diagrams show the proportion of community niche breadth variation explained by different factors for bacteria (a), fungi (b), and plants (c). Residuals indicate unexplained variance. SEM path diagrams for bacteria (d), fungi (e), and plants (f) show effects of environment and geography via community diversity (Shannon index) and community composition (NMDS) on community niche breadth. Numbers next to arrows are standardized path coefficients; *R*^2^ values represent variance explained for community niche breadth models.

Variation partitioning analysis (Fig. 4a–c) revealed that environmental factors were the primary drivers of the fungal community niche breadth, explaining 32.5% of the variation, whereas community diversity had a much smaller effect (8.4%). Bacterial and plant community niche breadths were mainly driven by biotic factors (13.2% and 15.2%, respectively), followed by environmental factors (12.8% and 14%). Geographic factors had minor effects in all three groups but, when combined with environmental and biotic factors, explained 33.1%, 5.8%, and 28.9% of the variation in niche breadth for bacteria, fungi, and plant communities, respectively. Compared with the VPA models that included only geographic and environmental factors, the explanatory power for community niche breadth markedly decreased (Fig. S3), with substantially higher residual variation in bacterial (22.3%), fungal (47.4%), and plant (40.4%) communities. Consistent with these kingdom-specific patterns, integrated analysis of overall niche breadth showed that incorporating biotic factors substantially improved the explanatory power and reduced residual variation from 0.391 to 0.244 (Fig. S5a and b). The shared contribution of environmental, geographic, and biotic factors explained a considerable proportion of the total variation (0.357), whereas geographic distance alone contributed relatively little to niche breadth variation.

The SEM for bacterial communities showed the highest explanatory power for niche breadth variation (*R*^2^ = 0.621; Fig. 4d–f, Table 1), whereas the models for fungal and plant communities had relatively lower explanatory power (*R*^2^ = 0.21 and 0.26, respectively), consistent with the VPA results, indicating bacterial community niche breadth is more directly explained by current environment and community composition, whereas plant and fungal community niche breadth are regulated by more complex mechanisms. Specifically, the niche breadth of all three groups was jointly driven by direct effects of environmental factors and indirect effects via community composition. Environmental factors had the strongest total effects on the bacteria (direct effect = 0.12; indirect effect = 0.35) and plant (direct effect = 0.18; indirect effect = 0.14) community niche breadths. Community composition (path coefficient = 0.44) and diversity (direct effect = 0.39; indirect effect = 0.18) also significantly positively influenced bacterial community niche breadth. Plant community niche breadth was directly driven by community diversity (direct effect = 0.26) and composition (direct effect = 0.26). Fungal community niche breadth was mainly governed by the environment and community composition (total environmental effect = 0.32; community composition = 0.34), with a small direct effect of community diversity (0.03) and a relatively larger indirect effect (0.19). Geographic factors had weak negative effects on community niche breadth across bacteria, fungi, and plant communities. Similarly, integrated SEM analysis based on the combined dataset further demonstrated that environmental dissimilarity positively affected both community diversity and composition and exerted both direct and indirect influences on niche breadth, whereas geographic distance had negligible effects on community composition and niche breadth (Fig. S6, Table 1).

**Table 1:** Standardized direct and indirect path coefficients estimated from SEM for niche breadth of bacterial, fungal, and plant communities. Direct effects indicate the influence of predictor variables directly on community niche breadth, while indirect effects are mediated via intermediate variables.

Community niche breadth		Environment	Geography	Diversity	Composition
Bacteria	Standardized Direct Effects	0.12	−0.02	0.39	0.44
	Standardized Indirect Effects	0.35	0.00	0.18	0.00
Fungi	Standardized Direct Effects	0.18	0.00	0.03	0.34
	Standardized Indirect Effects	0.14	−0.01	0.19	0.00
Plant	Standardized Direct Effects	0.18	−0.05	0.26	0.26
	Standardized Indirect Effects	0.14	−0.01	0.04	0.00
Overall	Standardized Direct Effects	0.20	−0.02	0.39	0.30
	Standardized Indirect Effects	0.26	−0.01	0.12	0.00

## Discussion

### Ecological strategies underlying cross-kingdom differences in niche breadth

Our study revealed clear, statistically significant differences in niche breadth among soil bacteria, fungi, and plants, with fungi exhibiting the broadest niche breadths, followed by bacteria, whereas plants showed the narrowest niches (Fig. 2). These contrasts likely reflect broad differences in life history strategies, modes of resource acquisition, dispersal capacity, and physiological adaptability among the three groups [64].

Soil fungal communities consistently exhibited broader mean niche breadths than soil bacterial communities. This pattern may be related to their high dispersal potential, trophic versatility, and capacity to persist under heterogeneous and stressful conditions. Fungi disperse efficiently via airborne spores across wide environmental gradients [64–66], and they can adopt multiple trophic strategies, including saprotrophy, symbiosis, and parasitism [67, 68]. Additionally, fungal hyphal growth may facilitate resource acquisition and persistence in structurally complex or oligotrophic soils [69, 70], collectively contributing to broader realized ecological breadths.

Soil bacterial communities exhibited somewhat narrower niche breadths. Our analyses showed that bacterial niche breadth was more strongly associated with community-level biotic factors, such as diversity and composition, than fungal niche breadth, suggesting a stronger dependence on community context during niche expansion. This context dependence may also contribute to variation in niche breadth under different community settings. Although bacteria possess traits such as rapid growth rates, high surface-area-to-volume ratios, and metabolic plasticity that favour opportunistic resource use [69, 71, 72], these traits may be less advantageous in the nutrient-poor, cold, and arid soils of alpine and desert grasslands. Under persistent environmental stress, bacterial communities may experience evolutionary pressures towards niche specialization. Furthermore, fungal antagonism—e.g. through antibiotic production or competitive exclusion—may impose additional constraints on bacterial expansion and functional potential [73–75], thereby limiting their realized niche breadth in shared habitats.

In contrast to our hypothesis, plant communities exhibited the narrowest mean niche breadth. This may be due to their sessile lifestyle, large body size, and dependence on abiotic conditions for resource acquisition via photosynthesis and root uptake. Such traits make plants particularly sensitive to spatiotemporal variation in water, nutrients, light, and temperature [76, 77], limiting their ability to adapt to the highly variable and harsh environments of the Qinghai–Tibet Plateau. This pattern aligns with previous studies indicating that plant niche breadth is shaped by interactions with soil properties and microbial communities [78–80]. Interestingly, despite their narrow average niche breadth, plants exhibited the greatest spatial variability in community mean niche breadth across sampling sites. This high variability likely reflects the strong dependence of plant species on local resource availability and the composition of neighbouring organisms [17, 81], as well as the influences of anthropogenic disturbance, successional stage, and vegetation type [82, 83]. These factors together contribute to a wide range of niche strategies among plant communities across the plateau.

### Differential responses of cross-kingdom niche breadth to environmental gradients

Bacterial, fungal, and plant communities exhibited significant variation in niche breadth in response to environmental gradients, but the dominant drivers and response patterns differed substantially across groups (Fig. 3c). Overall, microbial communities (bacteria and fungi) were more sensitive to local soil physicochemical properties, while plant communities responded primarily to macroclimatic factors. These differences likely reflect fundamental disparities in organismal size, life history traits, and environmental responsiveness. Microorganisms, with their small body size, short generation times, and high physiological plasticity, are particularly responsive to fine-scale resource heterogeneity and abiotic stressors.

Among microbial variables, SM emerged as the strongest predictor of bacterial niche breadth (Fig. 3c), likely due to its essential role in regulating microbial metabolism, dispersal, and survival [84–86]. In contrast, fungal niche breadth was jointly influenced by elevation, SM, and TC (Fig. 3c). As obligate heterotrophs, fungi depend heavily on carbon availability. TC, which reflects organic matter accumulation, supports diverse enzymatic and metabolic pathways, thereby enabling fungi to exploit a broader range of substrates and environmental conditions [87]. Fungi are also well adapted to physical and environmental stress, especially in alpine regions, owing to traits such as spore-based long-distance dispersal, hyphal growth, and enzymatic decomposition capabilities [69]. These traits likely underpin their capacity for niche expansion under fluctuating or extreme conditions. In contrast, plant niche breadth was most strongly influenced by MAP (Fig. 3c), which affects soil moisture regimes, nutrient diffusion, and root absorption efficiency. Unlike microorganisms, plants have limited mobility and delayed demographic responses, rendering them more constrained by broad-scale climatic variables than by local edaphic variation. Their ability to adjust to rapid microenvironmental changes is limited, reinforcing their dependence on precipitation and temperature regimes.

Elevation emerged as a particularly important integrative variable, encapsulating co-variation in temperature, moisture, radiation, and edaphic conditions [88]. For bacterial and fungal communities niche breadth increased with elevation—a pattern broadly consistent with Rapoport’s elevational rule (Fig. 3c), which posits that organisms at higher elevations tend to have broader environmental tolerances and wider distributions [89]. Alpine environments are typically characterized by low temperatures, strong diurnal temperature variation, limited nutrient input, and frequent abiotic stress [90, 91], all of which impose strong environmental filters that favour microbial taxa with broader ecological tolerances. Supporting this, Liu *et al*. [92] reported that elevational gradients shape both community assembly processes and niche characteristics in soil bacterial communities, with stronger environmental filtering at higher elevations. In contrast, plant niche breadth exhibited a unimodal (hump-shaped) response to elevation, with peak breadth observed at mid-elevations (Fig. 3c). This pattern likely reflects nonlinear responses to elevational stress, where mid-elevation zones offer more optimal climatic conditions—moderate temperature and precipitation—for plant growth and coexistence. These conditions likely promote species coexistence, functional diversity, and niche differentiation. At low elevations, plant communities may be dominated by slower-growing, disturbance-tolerant species with narrower niches [19, 42], while at high elevations, extreme abiotic stress (e.g. frost events, short growing seasons) selects for functionally similar taxa with specialized traits, leading to increased functional redundancy and reduced niche breadth [93].

### Interactive roles of abiotic filtering and community processes in shaping niche breadth

Our results indicate that community-level niche breadth is jointly shaped by abiotic filtering and community-level biotic context, rather than by environmental conditions alone. When niche breadth was explained using only environmental and geographic variables, a substantial proportion of variance remained unexplained (Fig. S3), suggesting that measured abiotic gradients captured only part of the processes structuring niche breadth. After incorporating biotic variables, the unexplained variance was markedly reduced (Fig. 4), indicating that community-level attributes such as diversity and composition account for additional variation in niche breadth. At the same time, these biotic effects should be interpreted cautiously as statistical correlates of community context, rather than as direct evidence of specific biological interactions, because unmeasured environmental factors may also indirectly appear as apparent biotic effects through their influence on community structure [94].

Against this joint abiotic–biotic framework, the relative importance of these two components differed among biological groups. In soil fungal communities, niche breadth was more strongly associated with abiotic environmental conditions (Fig. 4b), suggesting that fungal niche expansion is more directly constrained by environmental gradients. This pattern may be related to the strong resource acquisition capacity and trophic flexibility of fungi, which likely render their realized niche breadth particularly sensitive to environmental variation [67, 68]. However, this also leads to high intertaxon variability in environmental responses, rendering fungal niche breadth particularly sensitive to environmental gradients. In contrast, soil bacterial and plant communities exhibited niche breadth patterns that were more strongly associated with community-level biotic variables, particularly community composition (Fig. 4a and c). For plants, niche breadth may depend more strongly on local resource competition and co-occurring species combinations. Plants often reduce resource competition through mechanisms such as root stratification and temporal niche partitioning across growing seasons [95, 96], thereby making their niche breadth highly dependent on local community structure and neighbourhood context. By contrast, although soil bacterial communities can respond rapidly to environmental change, their community structure is often regulated by complex microbial interaction networks, including mutualism, competition, and antagonism [97, 98]. Consequently, bacterial niche breadth may depend more strongly on the stability and assembly background of microbial communities.

The role of community composition was particularly notable. In this study, community composition significantly influenced niche breadth across biological groups, suggesting that realized niche breadth is shaped not only by external environmental filtering but also by the biological context in which taxa are embedded. However, the ecological meaning of this pattern likely differs among groups. In bacterial communities, the association between niche breadth and community composition may reflect stronger sensitivity to short-term environmental dynamics and stochastic or deterministic community assembly processes. In contrast, plant niche breadth may be more strongly associated with long-term ecological stability, successional dynamics, and local species combinations. In stable ecosystems or late successional stages, plant communities are often dominated by narrow-niche specialists with efficient resource-use strategies, whereas under disturbed or highly heterogeneous conditions, generalist species with broader niches may gain competitive advantages [32, 42]. These findings were further supported by our SEM results (Fig. 4d–f). Collectively, these results suggest that community assembly plays a highly complex role in regulating niche breadth and may further influence the adaptability and stability of different biological groups under environmental change.

### Ecological implications of niche breadth for community stability and global change response

Niche breadth can be viewed as the functional occupation range of a community within environmental space, reflecting its overall capacity to utilize resources and respond to environmental variability [99]. It is therefore not only an outcome of community structure but also a key indicator of ecosystem stability and adaptive potential. Previous studies have shown that under strong biotic interactions, increases in species richness may lead to niche narrowing through enhanced niche differentiation [100–103]. However, at the community level, we observed a significant positive relationship between diversity and niche breadth (Fig. 4d–f), suggesting that in systems with high environmental heterogeneity or resource availability, increasing species diversity may expand the overall niche space through functional complementarity and resource partitioning [104]. Therefore, niche breadth is not governed solely by competition or environmental filtering but rather by the balance between these processes [105]. In this context, biodiversity loss may not only reduce species richness but also alter internal niche structure. In systems dominated by biotic interactions, declining diversity may weaken competitive constraints and modify niche differentiation processes, whereas in environmentally driven systems, it may lead to contraction of niche space, thereby reducing ecosystem adaptive capacity under environmental change [106].

In the context of global change, the mechanisms underlying niche breadth formation have important predictive implications [99, 107]. Our results indicate that in alpine grassland ecosystems, abiotic factors influence niche breadth not only through direct pathways but also indirectly via biotic processes such as community diversity and composition. This dual regulation mechanism is consistently observed across different biological groups, suggesting a certain degree of generality. Notably, these indirect pathways may be particularly susceptible to perturbation under global change. For example, as our results show that mean annual temperature and precipitation are key environmental drivers of niche breadth (Fig. 3a), changes in temperature and precipitation regimes may influence species distributions and community assembly processes, thereby reshaping niche structure and community adaptive capacity [26]. Therefore, predicting ecosystem responses to environmental change requires integrating both the direct effects of abiotic drivers and the cascading effects mediated through changes in community diversity and composition. On this basis, from the perspective of ecosystem management and biodiversity conservation, niche breadth and its distribution in environmental space not only reflect how communities respond to environmental change but also indicate the potential capacity of ecosystems to maintain key functions [99]. Therefore, focusing solely on species richness may be insufficient to fully assess ecosystem stability; instead, it is necessary to consider the coordinated changes in community structure and niche characteristics under environmental change. Maintaining high niche diversity and functional differentiation can enhance the buffering capacity of ecosystems against global change and improve their long-term stability and resilience.

## Conclusion

This study revealed distinct ecological strategies across biological groups in response to environmental gradients in world’s highest plateau. Fungal communities exhibited broader niche breadths than bacterial and plant communities, while plant communities displayed the highest variation in niche breadth across sites. Bacterial and plant niche breadths were more strongly regulated by biotic factors. In contrast, the fungal niche breadth was primarily driven by environmental factors, both directly and indirectly. Abiotic factors influence niche breadth directly and indirectly by shaping community diversity and composition. These findings emphasize the importance of biotic–abiotic interactions in determining community-level environmental adaptability, as well as specificity of fungal niche breadth pattern. We suggest niche breadth can serve as a valuable indicator for understanding how ecosystems respond to environmental changes. This cross-kingdom perspective deepens our understanding of the mechanisms underlying ecosystem stability and provides theoretical support for biodiversity conservation and management of alpine grasslands in the context of climate change.

## Supplementary Material

Supplementary_material_ycag193

## Data Availability

All 16S rRNA gene and ITS region sequences have been deposited in the European Nucleotide Archive (ENA) under accession numbers PRJDB2044 and PRJEB16010.
