# Supplementary material for: Contrasts in niche breadth and its drivers between soil microbes and plants in alpine grasslands
Source: ISME Commun. 2026 Jul 9;6(1):ycag193. doi: 10.1093/ismeco/ycag193 (PMC13529343; doi:10.1093/ismeco/ycag193)
Supplement: Supplementary file 1 [file supplementary_material_ycag193.zip › Suppleentary_materials_260804_ycag193.docx]

**Contrasts in niche breadth and its drivers between soil microbes and plants in alpine grasslands**

Lei Chen^1^, Ke Dong^2^, Mengchen Zhao^1^, Zhi Yu^3^, Nan Li^4^, Dorsaf Kerfahi^5^, Yu Shi^6^, Xin Jing^7^, Litong Chen^8^, Haiyan Chu^9,10^, Jinsheng He^11^, Sang-Seob Lee^1,*^, Teng Yang^9,10,12,*^, and Jonathan M. Adams^13,*^

^1^ Department of Integrative Biotechnology, Sungkyunkwan University, Suwon 16419, Republic of Korea

^2^ Department of Biological Sciences, Kyonggi University, Suwon 16227, Republic of Korea

^3^ Department of Environmental Health Sciences, Graduate School of Public Health, Seoul National University, Seoul 08826, Republic of Korea

^4^ Key Laboratory of Climate, Resources and Environment in Continental Shelf Sea and Deep Sea of Department of Education of Guangdong Province, Department of Oceanography, Key Laboratory for Coastal Ocean Variation and Disaster Prediction, College of Ocean and Meteorology, Guangdong Ocean University, Zhanjiang 524088, China

^5^ Department of Biological Sciences, Department of Natural Sciences, Keimyung University, Daegu 42601, Republic of Korea

^6^ State Key Laboratory of Crop Stress Adaptation and Improvement, School of Life Sciences, Henan University, Kaifeng 475004, China

^7^ State Key Laboratory of Herbage Improvement and Grassland Agro-Ecosystems, and College of Pastoral Agriculture Science and Technology, Lanzhou University, Lanzhou 730000, China

^8^ Qinghai Haibei National Field Research Station of Alpine Grassland Ecosystem, Northwest Institute of Plateau Biology, Chinese Academy of Sciences, Xining 810008, China

^9^ State Key Laboratory of Soil and Sustainable Agriculture, Institute of Soil Science, Chinese Academy of Sciences, Nanjing 211135, China

^10^ University of Chinese Academy of Sciences, Beijing 101408, China

^11^ Institute of Ecology, College of Urban and Environmental Sciences, and Key Laboratory for Earth Surface Processes of the Ministry of Education, Peking University, Beijing 100871, China

^12^ University of Chinese Academy of Sciences, Nanjing 211135, China

^13^ School of Geography and Ocean Science, Nanjing University, Nanjing 210023, China

**^*^Corresponding author:**

Sang-Seob Lee: [syhwh@skku.edu](mailto:syhwh@skku.edu); Department of Integrative Biotechnology, Sungkyunkwan University, Suwon 16419, Republic of Korea

Teng Yang: [tyang@issas.ac.cn](mailto:tyang@issas.ac.cn); State Key Laboratory of Soil and Sustainable Agriculture, Institute of Soil Science, Chinese Academy of Sciences, No. 298 Chuangyou Road, Nanjing 211135, China; University of Chinese Academy of Sciences, Beijing 100049, China; University of Chinese Academy of Sciences, Nanjing 211135, China.

Jonathan M. Adams: jonadams@nju.edu.cn; School of Geography and Ocean Science, Nanjing University, Nanjing 210023, China

**This file includes:**

1. Figures

2. Tables

**Figures**

Figure S1. PCA of all environmental variables across sampling sites. The length of each arrow indicates the contribution of the corresponding variable, while the angles between arrows represent the correlations among variables. Only the first two principal components are shown.

Figure S2. OTU-level niche breadths of bacteria, fungi, and plants. Significant differences among groups are indicated by different letters (*P* < 0.05).

Figure S3. Independent and joint effects of environmental and geographic factors on community niche breadth. Venn diagrams show the proportion of variance in niche breadth explained by different factors for bacterial (A), fungal (B), and plant (C) communities. "Residuals" represent the unexplained variance.

Figure S4. Variation in niche breadth at the phylum and genus levels across biological groups. Differences in niche breadth among major bacterial phyla (a) and fungal phyla (b), and among dominant plant genera (c). Different letters indicate significant differences among groups (P < 0.05).

Figure S5. Variation partitioning analysis of overall niche breadth based on abiotic and biotic factors. (a) Partitioning including only geographic and environmental variables. (b) Partitioning including geographic, environmental, and biotic variables. Numbers indicate the proportion of explained variation; residuals represent unexplained variation.

Figure S6. Structural equation model showing the direct and indirect effects of environmental, geographic, and biotic factors on overall niche breadth. Numbers on arrows represent standardized path coefficients. Model fit statistics are shown in the figure.

**Supplementary Tables**

**All supplementary tables are provided in an Excel table file.**

**Table S1.** Comparison of within-site and between-site Bray–Curtis dissimilarity across kingdoms. Values represent mean ± standard deviation (SD) of pairwise Bray–Curtis dissimilarity. Within-site dissimilarity was calculated among samples within the same site, whereas between-site dissimilarity was calculated among samples from different sites.

**Table S2.** Plant species composition and abundance across samples with taxonomic and functional attributes.

**Table S3.** Environmental and geographic variables measured for each sample.

**Table S4.** Bacterial OTU table with taxonomic classification and abundance across samples.

**Table S5.** Fungal OTU table with taxonomic classification and abundance across samples.

**Table S6.** Alpha diversity indices and NMDS coordinates of bacterial, fungal, and plant communities across samples.

**Table S7.** Variance explained by each principal component axis in the PCA based on all environmental variables.

**Table S8.** Overall niche breadth of bacterial OTUs and their niche positions and niche breadths along the first ten principal component (PC) axes.

**Table S9.** Overall niche breadth of fungal OTUs and their niche positions and niche breadths along the first ten principal component (PC) axes.

**Table S10.** Overall niche breadth of plant species and their niche positions and niche breadths along the first ten principal component (PC) axes.

**Table S11.** Community-level niche breadth of bacterial, fungal, and plant communities across samples.
